# Interleukin-1 Receptor-Associated Kinase-3 Is a Key Inhibitor of Inflammation in Obesity and Metabolic Syndrome

**DOI:** 10.1371/journal.pone.0030414

**Published:** 2012-01-17

**Authors:** Maarten Hulsmans, Benjamine Geeraert, Dieuwke De Keyzer, Ann Mertens, Matthias Lannoo, Bart Vanaudenaerde, Marc Hoylaerts, Nora Benhabilès, Christos Tsatsanis, Chantal Mathieu, Paul Holvoet

**Affiliations:** 1 Atherosclerosis and Metabolism Unit, Department of Cardiovascular Diseases, Katholieke Universiteit Leuven, Leuven, Belgium; 2 Experimental Medicine and Endocrinology Section, Department of Experimental Medicine, Katholieke Universiteit Leuven, Leuven, Belgium; 3 Division of Abdominal Surgery, Katholieke Universiteit Leuven, Leuven, Belgium; 4 Pneumology Section, Department of Pathophysiology, Katholieke Universiteit Leuven, Leuven, Belgium; 5 Centre for Molecular and Vascular Biology, Department of Molecular and Cellular Medicine, Katholieke Universiteit Leuven, Leuven, Belgium; 6 Department of Clinical Chemistry, School of Medicine, University of Crete, Heraklion, Greece; The University of Kansas Medical Center, United States of America

## Abstract

**Background:**

Visceral obesity is associated with the rising incidence of type 2 diabetes and metabolic syndrome. Low-grade chronic inflammation and oxidative stress synergize in obesity and obesity-induced disorders.

**Objective:**

We searched a cluster of molecules that support interactions between these stress conditions in monocytes.

**Methods:**

*RNA* expressions in blood monocytes of two independent cohorts comprising 21 and 102 obese persons and 46 age-matched controls were determined by microarray and independently validated by quantitative RT-PCR analysis. The effect of three-month weight loss after bariatric surgery was determined. The effect of *RNA* silencing on inflammation and oxidative stress was studied in human monocytic THP-1 cells.

**Results:**

*Interleukin-1 receptor-associated kinase-3* (*IRAK3*), key inhibitor of IRAK/NFκB-mediated chronic inflammation, is downregulated in monocytes of obese persons. Low *IRAK3* was associated with high *superoxide dismutase-2* (*SOD2*), a marker of mitochondrial oxidative stress. A comparable expression profile was also detected in visceral adipose tissue of the same obese subjects. Low *IRAK3* and high *SOD2* was associated with a high prevalence of metabolic syndrome (odds ratio: 9.3; sensitivity: 91%; specificity: 77%). By comparison, the odds ratio of high-sensitivity C-reactive protein, a widely used marker of systemic inflammation, was 4.3 (sensitivity: 69%; specificity: 66%). Weight loss was associated with an increase in *IRAK3* and a decrease in *SOD2*, in association with a lowering of systemic inflammation and a decreasing number of metabolic syndrome components. We identified the increase in reactive oxygen species in combination with obesity-associated low adiponectin and high glucose and interleukin-6 as cause of the decrease in IRAK3 in THP-1 cells *in vitro*.

**Conclusion:**

IRAK3 is a key inhibitor of inflammation in association with obesity and metabolic syndrome. Our data warrant further evaluation of IRAK3 as a diagnostic and prognostic marker, and as a target for intervention.

## Introduction

Chronic low-grade inflammation is now considered to have a pivotal role in the development of obesity and associated metabolic diseases such as insulin resistance, type 2 diabetes (T2DM) and the metabolic syndrome and cardiovascular disease [1], [2]. It is recognized that maladaptive production of various adipocytokines (e.g. adiponectin, resistin, visfatin, and leptin) and pro-inflammatory cytokines, such as tumor necrosis factor-α (TNFα) and interleukin (IL)-6 and IL-1, are implicated in the development of obesity-related systemic inflammation and insulin resistance. In particular, the plasma levels of the adipocytokine adiponectin are significant lower in obese individuals and have been associated with inflammation, insulin resistance and the development of cardiovascular disease. Adiponectin is present in the plasma in its full length, forming homomultimers, and as globular adiponectin, a shorter product formed by macrophage-dependent elastase cleavage [3]. Globular adiponectin appears to be responsible for most biological effects of adiponectin [4], [5]. The protective effect of adiponectin has been attributed to its anti-inflammatory action [6].

Consistent with their central role in coordinating innate immunity and inflammation, the toll-like receptor family (TLRs) and downstream transcription factor NFκB play critical roles in obesity-associated inflammation. In particular, TLR2 and TLR4 are highly expressed in macrophages and adipose tissue and can be activated by (saturated and oxidatively modified) fatty acids, elevated during obesity, resulting in NFκB-dependent differentiation with enhanced secretion of pro-inflammatory cytokines (e.g. TNFα) [7], [8]. Negative regulation of macrophage activation and cytokine secretion primarily occurs at the signaling level. Interleukin-1 receptor-associated kinase-3 (IRAK3; also referred to as IRAKM) is a kinase-deficient member of the TLR/IRAK family that has been shown to be an important negative regulator of TLR-mediated cell signaling [9]–[11]. IRAK3 negatively regulates signaling by preventing dissociation of IRAK1 and IRAK4 from MyD88 and formation of IRAK – TNF receptor-associated factor-6 (TRAF6) complexes. This protein has been shown to regulate critical aspects of innate immunity [12], [13]. IRAK3 expression is limited to cells of monocytic lineage [11] and is a major mediator of globular adiponectin-induced endotoxin tolerance in macrophages [14]. The role of this inhibitory protein in regulating key aspects of macrophage polarization during obesity-related inflammation and changes at its expression levels in obesity has not yet been defined.

Obesity is also increasingly recognized as an oxidative stress state, evidenced by the strong association between obesity and circulating oxidized LDL (ox-LDL), which is a systemic marker of oxidative stress [15]–[17]. In addition, reactive oxygen species (ROS) play an important role in macrophage-mediated immunity and the oxidation of specific epitopes, which at their turn are important targets of innate immunity [18], emphasizing the existence of a vicious circle between oxidative stress and inflammation in obesity [19].

Considering the clear role of macrophages in the propagation of inflammatory signals in adipose tissue, we wanted to identify deregulated genes in circulating monocytes of obese individuals and compare them with their expression pattern following weight loss. Therefore, we collected monocytes of obese individuals and performed microarray analysis followed by quantitative real-time PCR (qRT-PCR) analysis on their extracts. We identified the TLR2 signaling pathway as most deregulated canonical pathway with *IRAK3* as downregulated key inhibitor that is associated with obesity-associated metabolic syndrome and loss of protective action of adiponectin against cardiovascular disease.

## Results

### Study cohorts and metabolic parameters

The first cohort comprised 14 lean controls (29% male; age: 33±3 years, mean ± SEM) and 21 morbidly obese individuals (33% male; age: 39±3 years), without clinical symptoms of cardiovascular disease. Obese subjects in the first cohort had higher IL-6, high sensitivity C-reactive protein (hs-CRP), leptin and glucose levels, and lower adiponectin levels, indicating the presence of systemic inflammation. The higher levels of circulating ox-LDL indicated systemic oxidative stress. Furthermore, insulin and triglyceride concentrations were higher; HDL-cholesterol was lower. Obese individuals had higher systolic and diastolic blood pressure. Insulin resistance, calculated by a homeostasis model assessment (HOMA-IR), was 86% higher in obese subjects (Table 1A). A cluster of risk factors for cardiovascular disease and T2DM including raised blood pressure, dyslipidemia (elevated triglycerides and/or decreased HDL-cholesterol), raised fasting glucose, and central obesity have become known as the metabolic syndrome. A person qualifies for the metabolic syndrome with three abnormal findings out of five [20]. Four controls used in this study had 1 metabolic syndrome component; 1 had 2. Two obese patients had 1, 7 had 2, 5 had 3, and 7 had 4 metabolic syndrome components. Thus, 57% of the obese individuals had the metabolic syndrome. Finally, we also collected blood of the obese subjects three months after bariatric surgery. The blood characteristics after short-term weight loss are depicted in Table 1A. In aggregate, there was less systemic inflammation but no reduction in circulating ox-LDL. Triglycerides, HOMA-IR and adiponectin concentrations were restored to levels of lean persons (Table 1A).

**Table 1:** Characteristics and gene expressions before and after weight loss in obese patients (1^st^ cohort).

	Lean controls (n = 14)	Obese patients (n = 21)	
		Before weight loss	After weight loss
**A. Characteristics**			
Age (years)	33±3	39±3	39±3
BMI (kg/m^2^)	21±1	44±1***	36±1*** ^/^ $$$
Leptin (ng/ml)	8.7±1.4	65.6±8.0***	21.0±3.5** ^/^ $$$
Adiponectin (µg/ml)	10.9±1.8	3.9±0.6**	7.0±1.0$$$
Glucose (mg/dl)	83±2	111±7***	89±4$$$
Insulin (mU/l)	10.3±1.8	16.5±2.1**	6.3±0.8$$$
HOMA-IR	2.1±0.4	3.9±0.5**	1.8±0.2$$$
Triglycerides (mg/dl)	80±7	132±11***	99±8$$
LDL-C (mg/dl)	110±9	85±6*	92±3
HDL-C (mg/dl)	64±4	49±3**	47±2**
SBP (mmHg)	120±3	137±3**	118±1$$$
DBP (mmHg)	75±3	86±2**	62±1*** ^/^ $$$
IL-6 (pg/ml)	1.8±0.2	4.8±0.4***	3.4±0.4*** ^/^ $
Hs-CRP (mg/l)	0.49±0.10	5.65±1.13***	3.45±0.82** ^/^ $
Ox-LDL (IU/l)	50±5	71±4**	69±4**
**B. Gene expressions**			
*TLR2*	0.99±0.08	1.54±0.06***	1.02±0.09$$$
*IRAK3*	0.98±0.04	0.49±0.03***	0.79±0.05** ^/^ $$$
*TNFAIP3*	1.05±0.13	1.54±0.11***	1.03±0.11$$
*TNFα*	1.05±0.09	2.18±0.31***	1.06±0.12$$
*SOD2*	1.00±0.05	2.65±0.28***	1.91±0.19*** ^/^ $

Data shown are means ± SEM.

*P<0.05,

**P<0.01 and

***P<0.001 obese compared with lean controls;

$ *P*<0.05,

$$ *P*<0.01 and

$$$ *P*<0.001 compared with before weight loss;

Abbreviations: BMI, body mass index; C, cholesterol; DBP, diastolic blood pressure; HOMA-IR, homeostasis model assessment of insulin resistance; hs-CRP, high sensitivity C-reactive protein; ox-LDL, oxidized LDL; SBP, systolic blood pressure.

The second cohort consisted of 25 lean control and 102 successive obese women with (n = 62) and without T2DM (n = 40). T2DM was defined according to the American Diabetes Association. Obese women with T2DM were older (Table 2). Compared to non-diabetics, diabetics had higher glucose and insulin concentrations; their HDL-cholesterol was lower. Their LDL-cholesterol was also lower due to more frequent use of statins (78% vs. 30% of women without diabetes). This can also explain lower ox-LDL concentrations in diabetics. IL-6 and hs-CRP levels were not different between obese persons with and without T2DM. Sixty percent of diabetics were treated with an ACE-inhibitor, 51% with a beta-blocker, 15% with a calcium-antagonist, and 44% with aspirin. Seven controls had 1, and 5 had 2 metabolic syndrome components. Three obese women without T2DM had 1, 13 had 2, 14 had 3 and 10 had 4 metabolic syndrome components. Thus 60% of them had the metabolic syndrome. Four obese women with T2DM had 2, 12 had 3, 28 had 4, and 18 had all 5 metabolic syndrome components. Thus 94% of diabetics had the metabolic syndrome.

**Table 2:** Characteristics of obese women with and without T2DM for validation (2^nd^ cohort).

	Lean controls (n = 25)	Obese women (n = 102)	
		Without T2DM (n = 40)	With T2DM (n = 62)
Age (years)	44±2	48±2*	56±1*** ^/^ $$$
BMI (kg/m^2^)	22±0.3	37±1***	37±0.3***
Leptin (ng/ml)	5.8±0.6	58.0±9.0***	44±3.4***
Adiponectin (µg/ml)	9.8±0.91	5.9±0.63***	5.2±0.61***
Glucose (mg/dl)	87±3	102±5*	135±6*** ^/^ $$
Insulin (mU/l)	7.9±1.0	17.1±3.5***	36.3±5.2*** ^/^ $$
HOMA-IR	1.7±0.2	4.6±1.0**	17±2.0*** ^/^ $$$
Triglycerides (mg/dl)	79±6	126±11***	153±8***
LDL-C (mg/dl)	79±5	102±6*	77±4$$
HDL-C (mg/dl)	64±3	53±2**	45±2*** ^/^ $$$
SBP (mmHg)	127±3	134±3**	139±2***
DBP (mmHg)	72±2	82±8**	81±1***
IL-6 (pg/ml)	2.6±0.1	4.6±0.5***	5.5±0.4***
Hs-CRP (mg/l)	1.42±0.44	6.63±1.50**	6.79±0.98***
Ox-LDL (IU/l)	36±3	55±4***	46±2** ^/^ $

Data shown are means ± SEM.

*P<0.05,

**P<0.01 and

***P<0.001 obese compared with lean controls;

$ *P*<0.05,

$$ *P*<0.01 and

$$$ *P*<0.001 compared with obese women without T2DM;

Abbreviations: BMI, body mass index; C, cholesterol; DBP, diastolic blood pressure; HOMA-IR, homeostasis model assessment of insulin resistance; hs-CRP, high sensitivity C-reactive protein; ox-LDL, oxidized LDL; SBP, systolic blood pressure; T2DM, type 2 diabetes.

### Identification and validation of IRAK3 as potential key inhibitor of monocyte-related inflammation and oxidative stress

**Figure 1:**
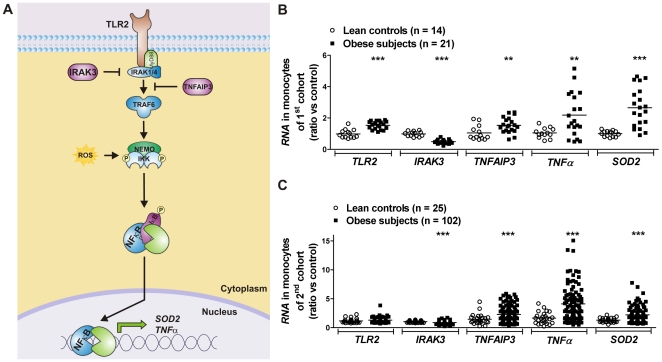
IRAK3 is a key inhibitor in monocyte-related mechanisms underlying inflammation and oxidative stress during obesity. (**A**) A structural model containing TLR2 as cell surface marker, NFκB as transcription factor, TNFα as inflammatory output, SOD2 as oxidative stress marker, and IRAK3 and TNFAIP3 as putative inhibitors was determined by promoter and gene annotation analysis of deregulated genes. Flow of the pathway at the protein interaction level is indicated by black arrows. Blunted arrows indicate inhibition. Phosphorylation is indicated by ???. Note that NFκB is constitutively bound to IκB molecules, which confine its localization to the cytosol. IKK complex phosphorylation of IκB promotes its degradation, thereby freeing NFκB to enter the nucleus and activate transcription of target genes. Gene expression of key molecules in the TLR2/NFκB inflammatory pathway was measured by qRT-PCR in blood monocytes of (**B**) the first cohort comprising 14 lean controls and 21 obese patients, and (**C**) the second cohort comprising 25 lean controls and 102 obese patients. Data are expressed as means. ^**^ *P*<0.01 and ^***^ *P*<0.001 obese persons compared with lean controls.

Microarray analysis of *RNA* extracts from monocytes of a subset of the first cohort (10 lean controls and 16 obese individuals) identified 512 differentially expressed genes. Ingenuity metabolic pathway analysis unveiled that the top five differentially regulated molecular and cellular functions were cell signaling, cell death, cellular growth and proliferation, cellular movement and cell-to-cell signaling. Related to signaling, genes involved in the TLR signaling, IL-6 and -10, circadian rhythm and PPAR signaling were frequently deregulated with the TLR2-related signaling pathway as most deregulated (11 out of 53 known key players were deregulated, *P* = 1.87×10^−5^). Structural modeling by promoter and gene annotation analysis of the deregulated genes in the TLR signaling pathway revealed a theoretical model (Figure 1A) containing TLR2 as cell surface marker, NFκB as transcription factor, TNFα as inflammatory output, SOD2 as oxidative stress marker, and IRAK3 and tumor necrosis factor alpha-induced protein-3 (TNFAIP3) as putative inhibitors of the TLR2/NFκB inflammatory pathway.

Figure 1B shows the expression of key molecules of the theoretical model in human blood monocytes of the first cohort. *IRAK3*, predominantly expressed in monocytes/macrophages, was the only inhibitor of which the expression was decreased in obese patients compared to lean controls and was associated with increased inflammation, evidenced by increased expression of *TLR2* and *TNFα*. Increased oxidative stress was further evidenced by decreased expression in genes involved in the oxidative defense (*SOD1* (-20%, *P*<0.001) and *catalase* (*CAT*, -13%, *P*<0.01)) as determined by qRT-PCR. However, these genes were not differentially expressed at a *P*-value<0.01 after microarray analysis.

To validate the observed expression profile, we determined the gene expressions in blood monocytes of an independent second cohort comprising 25 lean controls and 102 obese subjects (Figure 1C). *IRAK3* expression was decreased (-14%, *P*<0.001) and associated with increased *TNFα* and *SOD2* expressions. Gene expressions of the *IRAK3*-related pathway, except *TNFα*, were not different between obese patients with and without T2DM (Figure S1).

**Figure 2:**
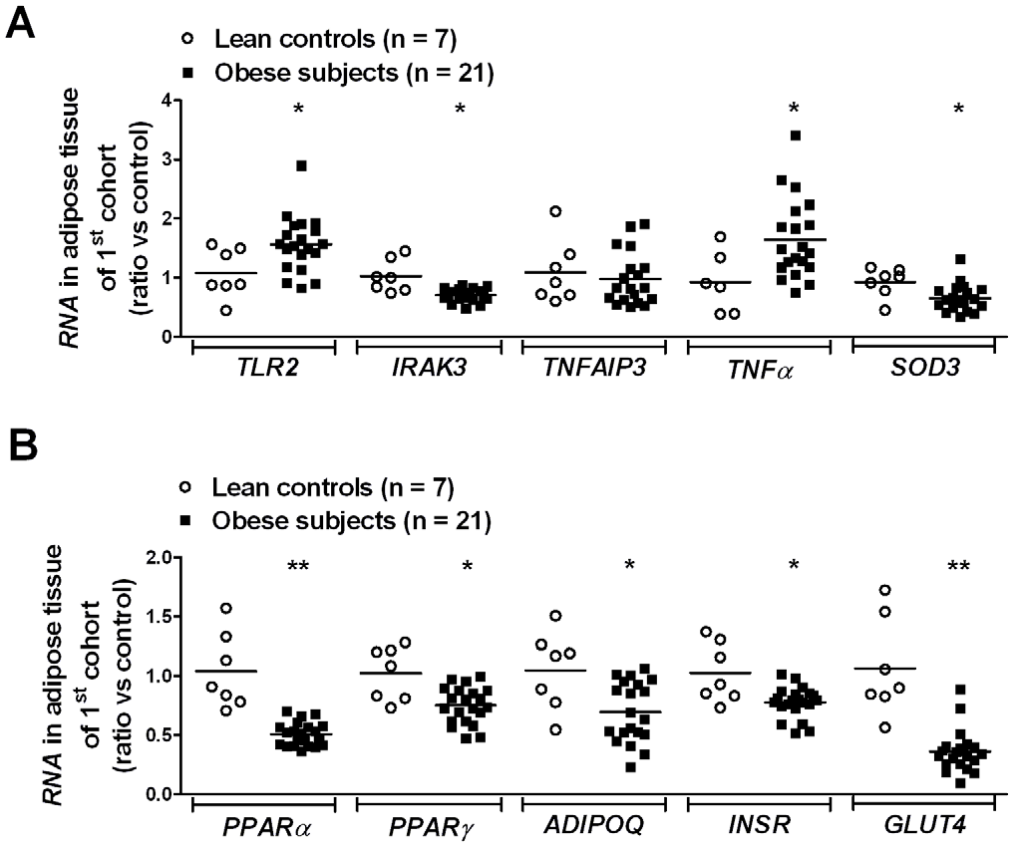
Gene expressions of the *IRAK3*-related pathway and adipocyte differentiation markers in visceral adipose tissue. (**A**) Gene expression in visceral adipose tissue was analyzed by measuring relative *RNA* levels using qRT-PCR for key molecules in the TLR2/NFκB inflammatory pathway. The adipose tissue specific antioxidant gene *SOD3* instead of *SOD2* was used as oxidative stress marker in visceral adipose tissue. (**B**) Relative *RNA* levels of markers of adipocyte differentiation (*PPARs* and *ADIPOQ*), insulin signaling (*INSR*) and glucose uptake (*GLUT4*) in visceral adipose tissue as determined by qRT-PCR. Data shown are means. ^*^ *P*<0.05 and ^**^ *P*<0.01 obese persons compared with lean controls; lean controls (n = 7), obese patients (n = 21).

Interestingly, a comparable expression profile was also detected in visceral adipose tissue of obese subjects compared with adipose tissue of lean controls (Figure 2A). Furthermore, markers of adipocyte differentiation (*PPARs*, *adiponectin* (*ADIPOQ*)), insulin signaling (*insulin receptor* (*INSR*)) and glucose uptake (*glucose transporter-4* (*GLUT4*)) were all decreased in adipose tissue of obese patients (Figure 2B). *IRAK3* correlated with *PPARα, PPARγ, ADIPOQ, INSR* and *GLUT4* (r_s_ = 0.66, 0.43, 0.43, 0.52 and 0.47 respectively; all *P*<0.05).

### Effect of short-term weight loss after bariatric surgery

Three-month weight loss after bariatric surgery was associated with increased *IRAK3* expression, and decreased inflammation and oxidative stress (Table 1B). In addition, *IRAK3* expression correlated negatively with systemic IL-6 (r_s_ = −0.40, *P*<0.01), ox-LDL (r_s_ = −0.32, P<0.05), and HOMA-IR (r_s_ = −0.42, *P*<0.01). It correlated positively with blood adiponectin (r_s_ = 0.28, *P*<0.05).

### Association with metabolic syndrome

Receiver operating characteristic curve analysis revealed that *IRAK3* (mean AUC, 95%CI: 0.63, 0.54–0.72, *P*<0.05), *TNFAIP3* (0.65, 0.56–0.73, *P*<0.01), *SOD2* (0.69, 0.60–0.77, *P*<0.001), and *TNFα* (0.71, 0.62–0.78, *P*<0.001) were associated with metabolic syndrome. *TLR2* was not.

Table 3 shows the odds ratios for metabolic syndrome in relation to *RNA* expressions in monocytes determined by Chi-square test with Yates' correction. Odd ratios of high *TNFAIP3*, or high *TNFα*, or high *SOD2*, or low *IRAK3* varied between 3 and 5. These values were comparable to that of hs-CRP, using the internationally accepted cut point of 3 mg/l. The value for the combination of low *IRAK3* or high *SOD2* was 6.9. The value increased to 9.3 when *IRAK3* was low and *SOD2* was high. Other combinations did not yield higher odds ratios than that of separate markers.

**Table 3:** Association of *RNA* expressions in monocytes and blood levels with occurrence of metabolic syndrome.

Gene	Cut point*	OR	Sensitivity (%)	Specificity (%)	PPV (%)	NPV (%)
High TNFAIP3	≥1.77	3.3 (1.5–7.2)	71	57	48	78
High *TNFα*	≥2.58	3.8 (1.8–8.2)	67	65	52	78
High *SOD2*	≥1.67	4.5 (2.0–9.9)	71	65	52	80
Low *IRAK3*	≤0.77	4.8 (1.5–14)	91	32	42	87
High CRP	≥3.0	4.3 (2.0–9.3)	69	66	53	79
Low *IRAK3* or high *TNFAIP3*	As above	4.0 (1.8–8.6)	62	71	54	77
Low *IRAK3* or high *TNFα*	As above	4.6 (2.1–10)	64	72	56	79
Low *IRAK3* or high *SOD2*	As above	6.9 (3.1–15)	69	76	61	82
High *TNFα* or high *SOD2*	As above	3.5 (1.7–7.6)	62	68	52	77
High *TNFα* or high *TNFAIP3*	As above	3.1 (1.5–6.7)	53	73	52	74
High *TNFAIP3* or high *SOD2*	As above	4.0 (1.8–8.6)	62	71	54	77
Low *IRAK3* and high *SOD2*	As above	9.3 (2.4–36)	91	77	61	85

*Cut points were determined by ROC curve analysis. Data are means (and 95% confidence intervals). Abbreviations: OR, odds ratio; NPV, negative predictive value; PPV, positive predictive value.

### Regulation of IRAK3 expression in THP-1 monocytes

**Figure 3:**
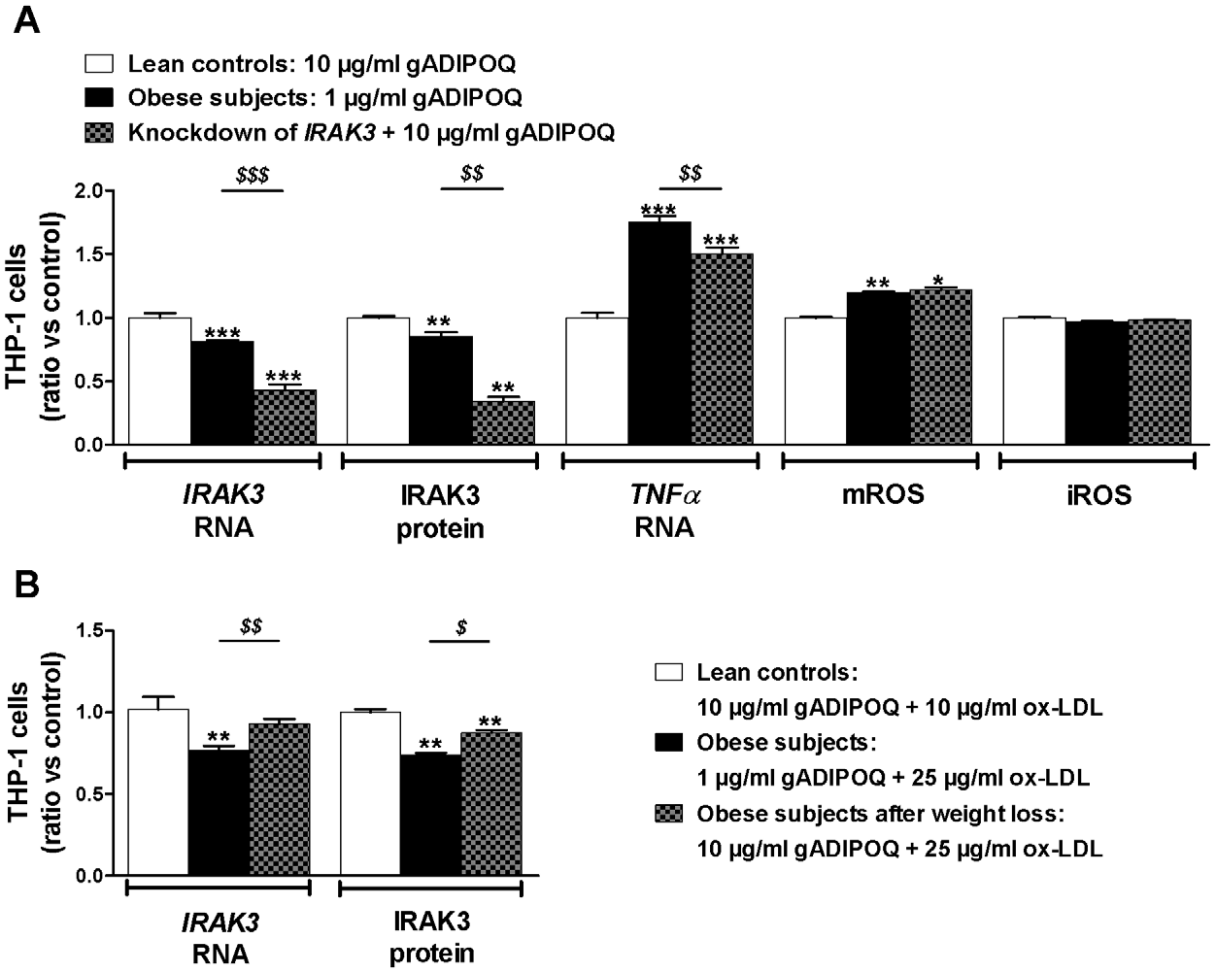
Regulation of IRAK3 expression in THP-1 monocytes. (**A**) Gene expression was analyzed by measuring relative *RNA* levels using qRT-PCR, protein expression and ROS production were determined by flow cytometry in THP-1 cells exposed to 1 or 10 µg/ml gADIPOQ (n = 6) or in *IRAK3*-depleted THP-1 cells exposed to 10 µg/ml gADIPOQ (n = 4) for 6 h and 24 h. Data shown are means ± SEM of 24 h exposed cells normalized to 6 h exposed cells. ^*^ *P*<0.05, ^**^ *P*<0.01 and ^***^ *P*<0.001 compared with THP-1 cells exposed to high gADIPOQ; ^$$^ *P*<0.01 and ^$$$^ *P*<0.001 compared with THP-1 cells exposed to low gADIPOQ. (**B**) Gene/protein expression in THP-1 cells exposed to 10 µg/ml gADIPOQ and 10 µg/ml ox-LDL (n = 6), 1 µg/ml gADIPOQ and 25 µg/ml ox-LDL (n = 6) or 10 µg/ml gADIPOQ and 25 µg/ml ox-LDL (n = 6). Data are expressed as means ± SEM. ^**^ *P*<0.01 compared with THP-1 cells exposed to 10 µg/ml gADIPOQ and 10 µg/ml ox-LDL; ^$^ *P*<0.05 and ^$$^ *P*<0.01 compared with THP-1 cells exposed to 1 µg/ml gADIPOQ and 25 µg/ml ox-LDL. Abbreviations: gADIPOQ, globular adiponectin, iROS, intracellular ROS; mROS, mitochondrial ROS; ox-LDL, oxidized LDL; ROS, reactive oxygen species.

To identify the mechanistic link between obesity and *IRAK3* depletion in circulating monocytes, we investigated the effect of high (as in lean controls) and low (as in obese persons) levels of adiponectin on IRAK3 expression. We determined the effect of globular adiponectin because this domain of the adiponectin protein appears to be responsible for most biological effects of adiponectin [3], [4]. Exposure of human monocytic THP-1 cells to low levels of adiponectin resulted in a decreased expression of IRAK3 (*RNA* and protein) compared to cells exposed to levels of adiponectin present in lean controls and obese persons after weight loss. This decrease was associated with more *TNFα* and mitochondrial ROS (mROS) production. Interestingly, the anti-inflammatory and anti-oxidative stress properties of high adiponectin were nullified when *IRAK3* was depleted by means of siRNAs (Figure 3A).

As shown above, circulating ox-LDL levels, also associated with metabolic disorders [17], were increased in obese patients, remained high after weight loss (Table 1A) and correlated negatively with *IRAK3* expression in monocytes (r_s_ = −0.32, *P*<0.05). The combined exposure of THP-1 cells to low adiponectin and high ox-LDL, as in obese patients, resulted in an additional decrease of IRAK3 (*RNA* and protein) compared to cells exposed to low adiponectin alone (Figure 3A and 3B). Interestingly, the IRAK3 expression in THP-1 cells exposed to high adiponectin combined with high ox-LDL levels, characteristic for obese patients after weight loss, resulted in an increased expression of IRAK3, thus emphasizing that adiponectin is essential to protect IRAK3 from inhibition by ox-LDL (Figure 3B).

**Figure 4:**
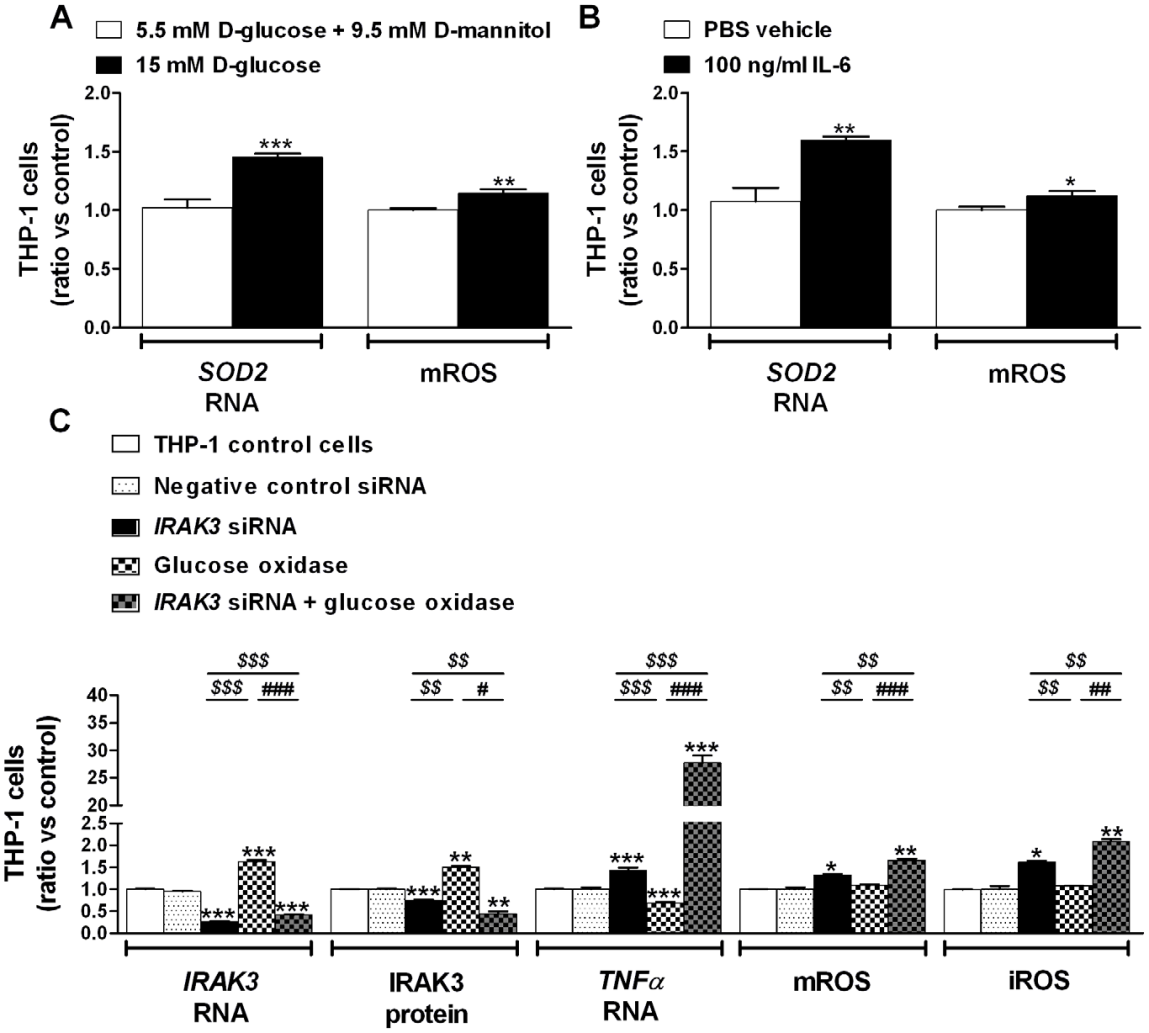
Exposure of IRAK3-depleted THP-1 cells to additional stress results in more inflammation and ROS. Gene expression was analyzed using qRT-PCR and mROS production was determined by flow cytometry in THP-1 cells exposed to (**A**) 5.5 mM D-glucose and 9.5 mM D-mannitol (osmotic control) or 15 mM D-glucose (n = 6), and (**B**) 100 ng/ml IL-6 (n = 6). Data shown are means ± SEM. ^*^ *P*<0.05, ^**^ *P*<0.01 and ^***^ *P*<0.001 compared with THP-1 cells exposed to 5.5 mM D-glucose or PBS vehicle. (**C**) Gene/protein expression and ROS production in THP-1 cells transiently transfected with siRNA targeting *IRAK3* (n = 10) or in THP-1 cells exposed to 100 mU/ml glucose oxidase with (n = 4) or without (n = 5) silencing of *IRAK3*. Data shown are means ± SEM. ^*^ *P*<0.05, ^**^ *P*<0.01 and ^***^ *P*<0.001 compared with THP-1 control cells or THP-1 cells transfected with negative control siRNA; ^$$^ *P*<0.01 and ^$$$^ *P*<0.001 compared with THP-1 cells transfected with *IRAK3* siRNA; ^##^ *P*<0.01 and ^###^ *P*<0.001 compared with THP-1 cells exposed to glucose oxidase. Abbreviations: iROS, intracellular ROS; mROS, mitochondrial ROS; ROS, reactive oxygen species.

Another blood component increased during obesity, decreased after short-term weight loss and able to induce oxidative stress in monocytes/macrophages, is glucose (Table 1A) [21]. Indeed, short-term exposure of THP-1 cells to high glucose, comparable to those in obese patients, resulted in increased mROS production despite an increased expression of *SOD2* (Figure 4A). Furthermore, similar results were produced after exposure of THP-1 cells to the cytokine IL-6, that is increased in obese subjects and decreased after weight loss (Table 1A and Figure 4B). We thus hypothesized that IRAK3-depletion together with an additional stress factor, such as oxidative stress, triggers more inflammation and oxidative stress in monocytes and adipose tissue macrophages. To test this hypothesis, we exposed *IRAK3*-depleted THP-1 cells to an external ROS source (glucose oxidase). Indeed, this resulted in more inflammation and ROS production compared with non-exposed *IRAK3*-depleted cells (Figure 4C).

## Discussion

Although recent evidence shows that a vicious circle of chronic inflammation and oxidative stress contribute to the development of obesity and associated metabolic diseases, common inhibitors of these processes have yet to be determined. Here, we identified IRAK3 as a key inhibitor of TLR2/NFκB-mediated chronic inflammation that is negatively associated with oxidative stress, and obesity-related insulin resistance and metabolic syndrome. We showed that *IRAK3* in monocytes is downregulated in obesity before the development of T2DM and cardiovascular disease and that its expression increased with weight loss. Interestingly, the combination of low *IRAK3* and high *SOD2*, a marker of mitochondrial oxidative stress was the strongest predictor of metabolic syndrome; it was even stronger than hs-CRP, the most widely used marker of systemic inflammation that was shown to be associated with metabolic syndrome [22], [23]. We further identified the mechanistic link between obesity and IRAK3 depletion in circulating monocytes by exposing human monocytic culture cells to variable concentrations of the obesity-associated hormone adiponectin. We showed that low adiponectin levels are responsible for the reduction in IRAK3 expression, which can be further reduced by exposing monocytes to additional stress factors such as ox-LDL. In addition, our results support a bidirectional relation between adiponectin and IRAK3: high adiponectin is required for upregulating IRAK3 in circulating monocytes and IRAK3 is required for adiponectin to display anti-oxidative and anti-inflammatory actions. The increased expression of *SOD2*, observed in activated blood monocytes of obese subjects, is possibly due to the increased systemic levels of IL-6 and glucose. The exposure to IL-6 and glucose in THP-1 cells was accompanied by increased mROS production despite the increased expression of *SOD2*. Indeed, glucose is a known substrate for ROS production in monocytes [21].

IRAK3 is an attractive candidate as inhibitor of monocyte-related innate immune responses since it is exclusively expressed in monocytes/macrophages and is a key inhibitor of TLR/NFκB signaling [11]. Indeed, several recent studies have demonstrated that IRAK3 regulates critical aspects of innate immunity, including the development of endotoxin tolerance and sepsis-induced alterations of antimicrobial responses [12], [13]. Furthermore, IRAK3 is cleaved in alveolar macrophages during pneumonia [24] and is an important regulator by which tumor-associated macrophages mimic the phenotype of alternatively activated (M2) macrophages [25]. However, the contribution of this inhibitory protein to activating and tolerizing circulating monocytes and macrophages during obesity-related inflammation has not yet been defined. To address this, we used microarray analysis to identify the most deregulated canonical pathway in circulating monocytes of obese individuals. Indeed, the TLR2 signaling pathway with IRAK3 as key inhibitor was highly deregulated in circulating monocytes and restored after short-term weight loss associated with decreased systemic inflammation. In addition, comparable changes in the *IRAK3* expression profile were detected in the visceral adipose tissue of obese subjects. This is in agreement with the finding that obesity is associated with enhanced activation of blood monocytes [26]. Thus, these results indicate that IRAK3 is a potential key inhibitor of chronic inflammation in blood monocytes and adipose tissue macrophages associated with obesity, and related metabolic disorders such as insulin resistance and metabolic syndrome.

Importantly, our *in vitro* data show that decreased systemic adiponectin concentrations due to impaired adipogenesis can lead to a decrease in monocytic IRAK3 expression. Indeed, low levels of adiponectin are responsible for the decrease in IRAK3 and this decrease can be amplified by additional exposure to systemic stress factors such as ox-LDL. However, IRAK3 expression depended on the adiponectin concentration rather than on the ox-LDL concentration further emphasizing the causal role of adiponectin in regulating IRAK3 expression in circulating monocytes. This is in agreement with the observations made after short-term weight loss and between obese persons with and without T2DM. Weight loss was associated with more systemic adiponectin and IRAK3 expression despite the higher circulating ox-LDL levels. Furthermore, obese patients with T2DM showed lower levels of ox-LDL but no increase in *IRAK3* expression. However, it is possible that there is a relation between local oxidative stress and IRAK3 expression. Indeed, we provided *in vitro* evidence that exposure of *IRAK3*-depleted THP-1 cells to an external ROS source, resulted in more inflammation and ROS production supporting the *in vivo* observation that IRAK3-depletion together with an additional stress factor enhances inflammation and oxidative stress.

Overall, these data support the hypothesis that not the reduction in adipose tissue per se, but the improved adipocyte differentiation and resulting increase in adiponectin is the mechanistic link with the changes in the expression of IRAK3 after weight loss. Interestingly, the anti-inflammatory and anti-oxidative action of high adiponectin was nullified when IRAK3 expression was depleted by means of siRNA supporting the recent finding that IRAK3 is a major mediator of the anti-inflammatory properties of adiponectin [14]. This observation is important in regard of adversative findings about the cardioprotective effect of adiponectin. Indeed, in recent studies higher adiponectin was associated with a significant increase in cardiovascular disease mortality in patients with metabolic diseases, and suggest that patients with low IRAK3 may not benefit from treatment with adiponectin or adiponectin mimetics [27], [28].

Therefore, such *RNA* expression analysis can be used to develop novel medical diagnostics for obesity-induced metabolic disorders and more particular to identify patients that will not benefit adiponectin treatment. Yet, the procedures for separating blood cells and extracting *RNA* are technically complicated and time consuming. However, these limitations may be overcome by using recently microfluidic lab-on-a-chip technology. This, indeed, represents a revolution in laboratory experimentation, bringing the benefits of miniaturization, integration and automation together. Currently, lab-on-a-chips are already being used for various applications in medicine and research, measuring specific types of cells and molecules in a patient's blood, and separating biological molecules for laboratory analyses.

In summary, we have demonstrated a bidirectional relation between adiponectin and IRAK3 in obesity. Low *IRAK3* in combination with high *SOD2* expression is a marker of the metabolic syndrome. In addition, the measurement of IRAK3 in monocytes can be important for the identification of obese patients that are more prone to adverse responses to adiponectin and adiponectin mimetics.

## Materials and Methods

### Materials

All chemicals were obtained from Sigma-Aldrich, unless stated otherwise. Polyclonal anti-IRAK3 antibody was purchased from Rockland and Alexa647-conjugated goat anti-rabbit IgG from Invitrogen. Human THP-1 monocytic cells (TIB-202) were obtained from ATCC.

### Patients and ethics statement

This study complies with the Declaration of Helsinki and the Medical Ethics Committee of the Katholieke Universiteit Leuven approved the study protocol. All human participants gave written informed consent. The first cohort comprised 14 lean control (27% male; WCF <80 cm) and 21 obese individuals (33% male; WCF: 128±11 cm, mean ± SEM). These 21 morbidly obese subjects were referred to our hospital for bariatric surgery. Before they were included, individuals were evaluated by an endocrinologist, an abdominal surgeon, a psychologist and a dietician. Only after multidisciplinary deliberation, the selected patients received a laparoscopic Roux-en-Y gastric bypass. A 30 ml fully divided gastric pouch was created and the jejunum, 30 cm distal of the ligament of Treitz, was anastomosed to it with a circular stapler of 25 mm. To restore intestinal transit, a fully stapled entero-entero anastomose was constructed 120 cm distal on the alimentary limb. In this way, the food passage was derived away from almost the whole stomach, the duodenum and the proximal jejunum [29]–[31]. The metabolic syndrome was defined according to the joint interim statement of 2009 [20]. The samples were collected between March 29^th^, 2005 and May 30^th^, 2006. The second group consisted of 25 lean control (WCF <80 cm) and 102 successive obese women with (n = 62; WCF: 115±2 cm) and without T2DM (n = 40; WCF: 123±5 cm). T2DM was defined according to the American Diabetes Association. The samples were collected at the Division of Endocrinology between September 27^th^ and December 20^th^, 2007. For analysis of the adipose tissue, we used the obese patients of the first cohort and lean controls (n = 7) that were referred to the hospital for minor surgical interventions. All participants were without symptoms of clinical atherosclerotic cardiovascular disease.

### Isolation of human monocytes

Blood samples were collected and after removal of the plasma fraction, peripheral blood mononuclear cells (PBMCs) were isolated using gradient separation on Histopaque-1077. Cells were washed three times in Ca^2+^- and Mg^2+^-free Dulbecco's (D)-PBS. PBMCs were incubated with CD14 microbeads (20 µl/1×10^7^ cells) for 15 min at 4°C. Cells were washed once and re-suspended in 500 µl Ca^2+^- and Mg^2+^-free DPBS containing 0.5% BSA/1 ×10^8^ cells. The suspension was then applied to an LS column in a MidiMACS Separator (Miltenyi) [32], [33]. We selected CD14^+^ monocytes because CD14 intensity expression on circulating monocytes was found to be associated with increased inflammation in patients with diabetes [34].

### Blood analysis

Human blood samples were centrifuged to prepare plasma samples for analysis. Total and HDL-cholesterol and triglyceride levels were determined with enzymatic methods (Boehringer Mannheim). LDL-cholesterol levels were calculated with the Friedewald formula. Insulin resistance was calculated by a homeostasis model assessment (HOMA) = fasting plasma insulin (mU/L) x fasting blood glucose (mM)/22.5. Plasma glucose was measured with the glucose oxidase method (on Vitros 750XRC, Johnson & Johnson), and insulin with an immunoassay (Biosource Technologies). Ox-LDL [35] (Mercodia), adiponectin, leptin and IL-6 were measured with ELISA (R&D Systems). Hs-CRP was measured on an Immage 800 Immunochemistry System (Beckman Coulter). Blood pressure was taken three times with the participant in a seated position after 5 minutes quiet rest. The average of the last two measurements was used for systolic and diastolic blood pressure. We tried to determine systemic TNFα levels in obese individuals but the interwell variation within ELISA plates was unacceptable. The coefficient of variance (%), defined as the standard deviation divided by the overall mean, was 41%.

### Cell culture

Human THP-1 monocytic cells were subcultured in RPMI 1640 (Gibco) as described previously in detail [36], [37]. For globular adiponectin incubation experiments, cells were cultured at a density of 1×10^6^ cells/ml in RPMI 1640 supplemented with 10% FBS and 5 µg/ml gentamicin. After 24 h, 1 or 10 µg/ml globular adiponectin (PeproTech) was added and the cells were incubated for 6 to 24 h. Globular adiponectin is a recombinant protein derived from human globular domain adiponectin cDNA expressed in *Escherichia coli*. This protein was endotoxin free (<2 EU/µg) according to the manufacturer. In addition, treatment of cells with globular adiponectin (10 µg/ml) in the presence of polymyxin B (50 µg/ml) did not affect globular adiponectin-related *TNFα* expression (data not shown). The ox-LDL incubation experiments were performed like previously described [36]. To induce ROS formation in THP-1 cells, the cells were incubated in RPMI 1640 containing 0.5% HSA in the absence or presence of 100 mU/ml glucose oxidase. For glucose incubation experiments, cells were cultured at a density of 1×10^6^ cells/ml in glucose-free RPMI 1640 supplemented with 10% FBS, 5 µg/ml gentamicin, and 5.5 mM D-glucose in a 5% CO_2_ incubator at 37°C. After 24 h, 9.5 mM D-glucose or 9.5 mM D-mannitol (osmotic control) was added and incubated for 24 h under normal growth conditions. For IL-6 experiments, THP-1 cells were stimulated with 100 ng/ml recombinant IL-6 (PeproTech) for 24 h.

To deplete *IRAK3 RNA*, THP-1 cells were transiently transfected with chemical synthesized HP GenomeWide siRNAs (Qiagen; human target sequence: 5′-CACATTCGAATCGGTATATTA-3′ (Hs_IRAK3_5) and 5′-CTGGATGTTCGTCATATTGAA-3′ (Hs_IRAK3_6)). As a negative control, we used AllStars Negative Control siRNA (Qiagen); as a positive control, we used Mm/Hs_MAPK1 control siRNA (Qiagen; target sequence: 5′-AATGCTGACTCCAAAGCTCTG-3′). Cells were transfected with 50 nM of siRNA using HiPerfect reagent (Qiagen), according to the manufacturer's instructions with some modifications. Briefly, THP-1 cells were seeded at a density of 1.5×10^5^/well (24-well plate) in 100 µl of growth medium. Next, HiPerfect/siRNA complexes (3 pmol of siRNA plus 6 µl of HiPerfect reagent) were formed in 0.1 ml of serum-free RPMI-1640 for 10 min at room temperature and then added to each well. After 6 hours of incubation under normal growth conditions, 400 µl of growth medium was added to each well and the cells were incubated for 42 hours. Gene silencing was monitored at the *RNA* level by means of qRT-PCR and at the protein level using flow cytometry. *IRAK3*-depleted THP-1 cells were exposed to globular adiponectin or glucose oxidase as mentioned above.

Cell viability, as determined by trypan blue exclusion, was >80%. mROS and iROS formation were measured with MitoSOX and CM-H_2_DCFDA. Cells were incubated with PBS containing 5 µM MitoSOX or 5 µM CM-H_2_DCFDA for 30 minutes at 37°C and 5% CO_2_. The labeled cells were washed twice with PBS and then suspended in warm PBS for analysis by flow cytometry (Becton, Dickinson and Company). For analysis of IRAK3 protein expression, a total of 2×10^5^ treated THP-1 cells were blocked with FcR blocking reagent (Miltenyi) for 10 min, followed by fixation and permeabilization using a fixation/permeabilization kit (Miltenyi), according to the manufacturer's instructions. Next, cells were incubated with 2.5 µg/ml anti-IRAK3 antibody or isotype-matched control IgG at room temperature for 30 min. After washing, cells were incubated with 1 µg/ml Alexa647-conjugated goat anti-rabbit IgG for another 30 min at room temperature. Finally, the cells were washed twice, and analyzed by flow cytometry (Becton, Dickinson and Company).

### RNA isolation, microarray and quantitative RT-PCR analysis

Total *RNA* was extracted with TRIzol reagent (Invitrogen) and purified on RNeasy Mini Kit columns (Qiagen). The *RNA* quality was assessed with the RNA 6000 Nano assay kit using the Agilent 2100 Bioanalyzer.

Microarray analysis of *RNA* expression was performed with Illumina's Sentrix Human-6 v2 Expression BeadChip Kit containing 46,713 probes/array targeting genes and known alternative splice variants from the RefSeq database release 17 and UniGene build 188. *RNA* was labeled, hybridized and scanned according to Illumina GLP standards by Aros AB laboratory. The raw data were normalized with the rank-invariant method (Illumina BeadStudio V2). This method uses a linear scaling of the populations being compared. The scaling factor is determined by rank-invariant genes. “Rank-invariant” genes are those genes whose expression values show a consistent order relative to other genes in the population. Of the 46,713 transcripts, 512 transcripts, which were mapped in the Ingenuity Pathway Analysis (IPA) program 5.5-802, were differentially expressed in monocytes of obese patients compared to lean controls at a *P*-value<0.01. Networks were built by means of the “Connect” and the “Path explorer” tool in IPA. Canonical pathways were derived using the “Analysis” tool in IPA. In order to build the most representative structural network starting from the deregulated genes in the TLR signaling pathway, we used the GEMS MatInspector, Frameworker, Gene2 promoter, and ElDorado software (Genomatix). The significance of the association between the dataset and the canonical pathway was measured in 2 ways. First, a ratio of the number of genes that were differentially expressed in monocytes of obese patients and were assigned to a particular signaling pathway to the total number of genes that belong to this signaling canonical pathway and were present on the microchip. Second, the Fischer's exact test was used to calculate a *P*-value determining the probability that the association between the differentially expressed genes in the dataset and the assigned genes in the canonical pathway is not explained by chance alone. The threshold was *P* = 0.01. The data discussed in this publication have been deposited in NCBI's Gene Expression Omnibus [38] and are accessible through GEO Series accession number GSE32575 (http://www.ncbi.nlm.nih.gov/geo/query/acc.cgi?acc=GSE32575).

qRT-PCR is a commonly used validation tool for confirming gene expression results obtained from microarray analysis. First-strand cDNA was generated from total *RNA* with the SuperScript VILO cDNA synthesis kit (Invitrogen). qRT-PCR was performed on a 7500 Fast Real-Time PCR system using Fast SYBRGreen master mix, according to the supplier protocols (Applied Biosystems). Oligonucleotides (Invitrogen) used as forward and reverse primers were designed using the “Primer Express” software (Applied Biosystems) and are summarized in Table S1. *RNA* expression levels were expressed as the ratio compared to controls as previously described [36], [37].

Note that microarray and qRT-PCR data often result in disagreement. It is well documented that both qRT-PCR and microarray analysis have inherent pitfalls that may significantly influence the data obtained from each method [39]. One of the microarray-related pitfalls is the fact that some oligonucleotide probes imprinted on the slide target the wrong gene [40]. One of the important disagreements between microarray and qRT-PCR analysis, was that the first identified IRAK3 as upregulated, whereas the latter identified it as downregulated. To make sure that primer sequences, used in qRT-PCR, target the right gene, their specificity was validated by Basic Local Alignment Search Tool (BLAST) [41]. Furthermore, cDNA clones (OriGene) for IRAK3 (TNFAIP3 and SOD2) were used to double check the primer specificity. In addition, PCR fragments were validated for GC/AT ratio, length, and amplification specificity with dissociation curve analysis and agarose gel electrophoresis [42].

### Statistical analysis

Lean and obese subjects were compared with an unpaired t-test with Welch's correction; obese subjects before and after weight loss were compared with a paired t-test (two-tailed); *in vitro* data were compared with the Mann–Whitney *U* test (GraphPad Prism 5). Correlations were calculated using the nonparametric Spearman's correlation coefficient (r_s_). Receiver operating characteristic curve (ROC) analysis was performed with MedCalc statistical software for biomedical research. Odds ratios were determined by Chi-square test with Yates' correction (GraphPad Prism 5). A *P*-value of less than 0.05 was considered statistically significant.

## Supporting Information

Figure S1 **Gene expression profile of obese women with and without T2DM.** Gene expression of key molecules in the TLR2/NFκB inflammatory pathway in blood monocytes of 25 lean controls, 40 obese women without T2DM and 62 obese women with T2DM. Data are expressed as means. ^*^ *P*<0.05, ^**^ *P*<0.01 and ^***^ *P*<0.001 obese compared with lean controls; ^$^ *P*<0.05 obese with T2DM compared with obese without T2DM; Abbreviation: T2DM, type 2 diabetes.
(TIF)

Table S1 **Primers used in qRT-PCR.**
(DOC)
