# Supplementary material for: Interleukin-1 Receptor-Associated Kinase-3 Is a Key Inhibitor of Inflammation in Obesity and Metabolic Syndrome
Source: PLoS One. 2012 Jan 17;7(1):e30414. doi: 10.1371/journal.pone.0030414 (PMC3260289; doi:10.1371/journal.pone.0030414)
Supplement: Table S1 — Primers used in qRT-PCR. (DOC) [file pone.0030414.s002.doc]

**Table S1. Primers used in qRT-PCR.**

| **Gene** | **Forward primer** | **Reverse primer** |  |
| --- | --- | --- | --- |
| ***ACTB*** | 5’-GGACCTGACCGACTACCTCATG-3’ | 5’-CGACGTAGCAGAGCTTCTCCTT-3’ |  |
| ***ADIPOQ*** | 5’-ATGCCCCAGCAAGTGTAACC-3’ | 5’-TCAGAAACAGGCACACAACTCA-3’ |  |
| ***CAT*** | 5’-CATCCAGAAGAAAGCGGTCAA-3’ | 5’-TCAGCATTGTACTTGTCCAGAAGAG-3’ |  |
| ***GLUT4*** | 5’-GGACAGGAGACAAGAAATCCAGTT-3’ | 5’-GCGTGGCAAGAATTCAGTGA-3’ |  |
| ***INSR*** | 5’-TGTGTACCTCTTGTGGCGTTTC-3’ | 5’-CTCAGTGCACCTCTCTCTTACATTG-3’ |  |
| ***IRAK3*** | 5’-TGCAACGCGGGCAAA-3’ | 5’-TTTAGTGATGTGGGAGGATCTTCA-3’ |  |
| ***PPARα*** | 5’-CAGCCTGTGGCCTCTGTAGTTAG-3’ | 5’-CCCTTTACACAACCGAAGTTCCT-3’ |  |
| ***PPARγ*** | 5’-ATGCAGGCTCCACTTTGATTG-3’ | 5’-CCAGTGGTTGCAGATTACAAGTATG-3’ |  |
| ***SOD1*** | 5’-TTGGGCAAAGGTGGAAATGA-3’ | 5’-CACCACAAGCCAAACGACTTC-3’ |  |
| ***SOD2*** | 5’-TGGAAGCCATCAAACGTGACT-3’ | 5’-TTTGTAAGTGTCCCCGTTCCTT-3’ |  |
| ***SOD3*** | 5’-CGGGAGTCTCAGGGCTTATG-3’ | 5’-GCCTCCATTTGTACCGAAACA-3’ |  |
| ***TLR2*** | 5’-TGCAAGTACGAGCTGGACTTCTC-3’ | 5’-GTGTTCATTATCTTCCGCAGCTT-3’ |  |
| ***TNFAIP3*** | 5’-TCCCTGCTCCTTCCCTATCTC-3’ | 5’-ATGTTTCGTGCTTCTCCTTATGAA-3’ |  |
| ***TNFα*** | 5’-CAAGCCTGTAGCCCATGTTGTA-3’ | 5’-TTGGCCAGGAGGGCATT-3’ |  |
